# The SPLASH/ICPC integrity marathon in Ibadan, Nigeria: incidence and management of injuries and marathon-related health problems

**DOI:** 10.1186/2052-1847-5-6

**Published:** 2013-04-15

**Authors:** Omoyemi O Ogwumike, Ade F Adeniyi

**Affiliations:** 1 Department of Physiotherapy, College of Medicine, University of Ibadan, Ibadan, Nigeria

**Keywords:** Physiotherapy, Marathon, Injuries, Runners

## Abstract

**Background:**

The growing interest in marathon runners and marathons in Nigeria has not been reflected in reports of injuries and other health problems associated with these events. This study therefore outlines the incidence of injuries, marathon-related health problems and delivery of physiotherapy at the maiden and second editions of the Splash 105.5 FM/ICPC Integrity Marathon in Ibadan city, south-west Nigeria in 2009 and 2010.

**Methods:**

Using a data entry sheet, demographics and information on running experience, past and present injuries and other health problems reported en route and at the finish line by the runners were documented. The prevalence of injuries and other health problems reported by previous and first-time runners were compared.

**Results:**

In both events, 16.3% and 17.2% of the runners respectively reported injuries with significant occurrence in first-time runners (p = 0.003 for 2009; p = 0.002 for 2010) mostly at the finish line. The reported injury type and site were muscle cramps and the thigh (39.7% and 76.4% respectively). Heat exhaustion was reported by 42.8% of runners in 2009 and 56.3% in 2010. Cryotherapy was mostly used in combination with other physiotherapy modalities in both years.

**Conclusion:**

Most of the injuries and other health problems were reported by first-time marathon runners mainly at the finish line. The most reported site of injury was the thigh while muscle cramps and heat exhaustions were the most reported types of injuries and health problems. First-time marathon runners should be adequately informed of the predisposition to injury during marathons and adequate body conditioning should be emphasized. Ample preparation and effective involvement of the physiotherapy team is essential for management of injured runners en route and at the finish line in a marathon.

## Background

During the last two decades, there has been a rising trend in marathon events and marathon runners [1]. Various marathon competitions are now being organised in sub-Saharan Africa, where these events were hitherto unpopular and where adequate manpower and infrastructure may not have been available to handle such events. Marathon runners usually include elite athletes and first-time runners, therefore the incidence and management of injuries in these runners cannot be overlooked [1,2].

Running, due to its high impact nature is associated with many injuries, especially in the lower extremities, some of which include: runner’s knee or chondromalacia patella, shin splints, muscle cramps especially the hamstrings, ankle sprains, iliotibial band syndrome, plantar fasciitis, achilles tendonitis, foot blisters and stress fractures. These injuries are usually caused by repetitive stress on the same tissues without enough time for recovery or lack of adequate muscle conditioning [2-5]. Generally few studies have been published on recent marathon events. Available reports on previous marathon races are from developed countries where such events are popular. There is therefore an observable want of published findings on the incidence and management of injuries during marathon events held in a developing country such as Nigeria.

The Splash 105.5 FM/ICPC marathon is organized annually by Splash FM, a private radio station in Ibadan south-west Nigeria with ICPC-the Independent Corrupt Practices Commission an anti-corruption agency set up by the Federal Government of Nigeria. The marathon is to celebrate the anniversary of the radio station and sensitize the populace about the fight against corruption in the Nigerian society. The two marathon events covered in this study took place on the 29^th^ and 21^st^ March 2009 and 2010 respectively. These events attracted several runners from many states some of whom were elite runners. This report presents the cases of injuries and other health problems that occurred at both events and were managed by physiotherapy.

## Methods

Eight physiotherapists and two physiotherapy assistants volunteered to help in provision of on-field paramedical services during the competitions. Also in attendance were medical officers who managed other medical conditions that occurred during the event. Preparations were made for medical emergencies that could arise. The physiotherapists had a number of meetings with other members of the medical team including the officials of the Zonal National Sports Commission. Planning of physiotherapy service considered the nature of the route course and the expected weather conditions.

The months of October to March are usually very hot months in the tropics. The average peak temperature three days before the event in Ibadan city was 32°C for 2009 and 34°C for 2010. These were above the wet bulb globe temperature range of 23-28°C which is already classified as high risk for distance running by the International Marathon Medical Directors Association [6]. In view of this, adequate arrangements were made for water points and light refreshments every 2-3 km along the marathon route to forestall dehydration and reduce heat injury [6,7]. No traffic except ambulances were allowed along the marathon routes to ensure free movement of the runners. Informed consent was obtained from the injured participants after which the age, sex, nature of complaints, type and site of injury and relevant past history of injuries were recorded in a prepared data entry sheet. Results for both years were compared using frequencies, percentages and chi-square analysis. Other medical problems were promptly handled by medical officers. The races commenced at 7.25 a.m. in 2009 and 7.42 a.m. in 2010.

## Results

The number of runners declined from 600 in 2009 to 320 in 2010 even though more males participated in both years (95.7% and 88.1%) respectively. The age range of runners was 17 to 37 years, mean age being 23.83 ± 3.49 years in 2009 and 26.67 ± 7.56 years in 2010. In 2009, 98 runners (16.3%) were managed for various injuries and other health problems sustained mostly by males (82.6%) (Table 1). Similarly in 2010, 17.2% of the runners were treated for various complaints using physical therapy. In both years, a significant difference occurred in the prevalence of injuries among those who have had previous experience of a marathon, previous experience of other racing events and those who were first-time marathon runners (p = 0.003 for 2009 and p = 0.002 for 2010). In both years, most of the runners who sustained injuries were first-time marathon runners without previous experience of any long distance racing competitions (60.2% in 2009 and 52.7% in 2010). More health problems were attended to at the finish line (64.3% in 2009 and 58.2% in 2010; p < 0.05) than along the marathon route.

**Table 1:** Statistics of the runners

**Characteristics**	**2009 Marathon**	**2010 Marathon**
	N=600	N=320
	**N (%)**	**N (%)**
***Total number of runners (N)***		
Male	574 (95.7)	282 (88.1)
Female	26 (4.3)	38 (11.9)
	**n (%)**	**n (%)**
***Number of injured runners (n)***	98 (16.3)	55 (17.2)
Male	81 (82.6)	48 (87.3)
Female	17 (17.4)	7 (12.7)
***Previous racing experience of injured runners***		
None	59 (60.2)	29 (52.7)
Marathon	15 (15.3)	12 (21.8)
Other races (12 km, 18 km & 21 km)	27 (27.5)	14 (25.5)
χ^2^	31.54	39.82
p-value	0.003	0.002
***Location where injury was treated***		
En route	35 (35.7)	23 (41.8)
Finish line	63 (64.3)	32 (58.2)
χ^2^	22.21	17.92
p-value	0.003	0.02

Key: N = Total number of runners, n= Number of injured runners, %= percentage of each variable i.e N and n.

**Figure 1:**
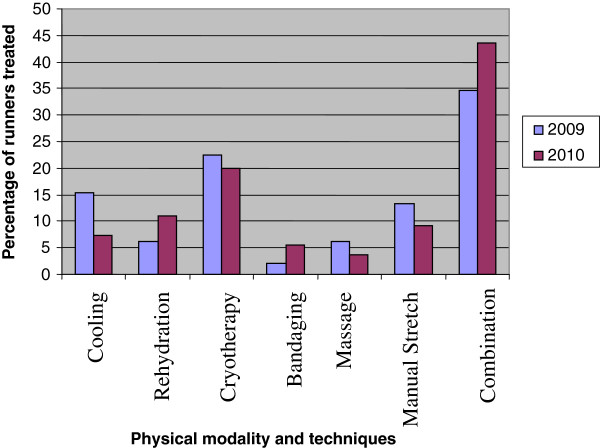
Treatment of injuries and other health problems using physical modalities and techniques.

Some runners reported injuries in only one part of the body while others reported in several parts. The most reported single site of injury was the thigh (39.7% and 76.4%) respectively for both years (Table 2). The groin was the least (9.2%) reported site of discomfort in the 2009 race while one runner (1.8%) reported shoulder pain in 2010. Overall, 37.8% and 49.1% of the injuries that occurred in 2009 and 2010 respectively were muscle cramps, followed by ligamentous sprains (25.5% and 41.8%) respectively. Heat exhaustion was the commonest health problem reported by the runners in both years (42.8% in 2009 and 56.3% in 2010). Injury exacerbation occurred in 38.2% in 2010 compared to 17.3% in 2009. Applied physiotherapy modalities and treatment techniques are as presented in Figure 1. Some received one modality of treatment while most (34.7% in 2009 and 43.6% in 2010) received a combination of physiotherapy modalities or techniques.

**Table 2:** Injuries and marathon-related health problems recorded in the first and second marathon events

	**2009 Marathon**	**2010 Marathon**
***Site of injury***	**n (%)**	**n (%)**
Thigh	39 (39.7)	42 (76.4)
Groin	9 (9.2)	2 (3.6)
Knee	28 (28.6)	7 (12.7)
Calf	19 (19.4)	26 (47.3)
Ankle	14 (14.3)	31 (56.4)
Foot	25 (25.5)	11 (20.0)
Shoulder	0 (0.0)	1 (1.8)
***Type of Injury***		
Toe blisters	22 (22.4)	10 (18.2)
Contusion	24 (24.5)	23 (41.8)
Sprain	25 (25.5)	23 (41.8)
Tendonitis	11 (11.2)	18 (32.7)
Muscle cramps	37 (37.8)	27 (49.1)
***Other health problems***		
Heat exhaustion	42 (42.8)	31 (56.3)
Hypothermia	11 (11.2)	9 (16.4)
***Injury Exacerbation***		
Yes	17 (17.3)	21 (38.2)
No	81 (82.6)	34 (61.8)

Key: n= frequency of injury or health problem, %= percentage occurrence.

## Discussion

This report examines the injuries and health problems that were treated using physical therapy at the Splash FM/ICPC Integrity marathon races in 2009 and 2010 in Ibadan, Nigeria. More males participated in both events and they had more injuries than the females. Both events recorded injuries in nearly one fifth of the runners. A broader comparison of our data could not be made with previous marathons in Nigeria or any other African country because published reports on these were not available. However, it was interesting to note that the prevalence of injuries and marathon-related health problems was significantly higher in first-time marathon runners than the elite runners. The predisposition to greater risk in the first-time marathon runners has been attributed to the fact that they are not elite athletes. Most of these first-time runners lacked awareness of and proper body conditioning necessary for a marathon race [8]. Some of them may have been overweight or underweight and may have over-trained without adequate strength training and probably lacked core muscle strength [2]. This underscores the importance of consistent participation to adequate conditioning of athletes for such events.

Marathon events have been described as fashionable endurance competitions in which thousands of participants from all age groups compete, some even after relatively little training [2]. Hence the 2009 Splash Marathon being the first of its kind in one of the largest cities in West Africa, attracted many participants. However, by the second event, the number of participants dropped by almost 50%. This may be due to the fact that it was no longer a novel event.

Reports of injuries at the finish line were significantly higher than during the race. This may be because most of the injured runners did not want to lose any time, since the winner of the race would have hefty money prize. However, most of those who reported injuries at the finish line confirmed injury onset during the course of the race. They probably were able to reach the finish line by sheer perseverance.

The exceptional capability of humans to run long distances under hot arid conditions has been linked to the use of a suite of anatomical, physiological and behavioural features which are uniquely found in humans. The specialised ability of cardiorespiratory endurance system permits humans to store and release energy effectively during a race and helps to keep the body’s centre of mass stable and overcome the thermoregulatory challenges of long distance running through sweating, evaporation and cooling [9].

Considering the site and type of injury, the part of the body that was reported the most was the thigh while the nature of discomfort mostly reported for both years was muscle cramps. The thigh as the commonest injury site could be due to chafing usually caused by repetitive rubbing of one area of skin against another or against an article of clothing. This occurs mostly in the upper thigh during running [2]. Considerable amounts of injury were also reported in the knee, calf, ankle and feet. This is in line with the report of a previous study, in which more than 90% of injuries in runners are recorded in the lower extremities, equally affecting the regions of the knee, shank, and foot [3]. This may be because of the weight-bearing nature of the joints and the repetitive movements that are involved in a full marathon race. Running has been shown to load the joints of the lower extremities with vertical forces that are 4 to 8 times greater than when walking [4]. For instance, a 70-kg runner sustains an average vertical force of 2800 tons acting on the hip, knees, and ankle joints over a marathon distance of slightly more than 42 km [10]. In addition, when muscle fatigue sets in near the end of the race, the bones and joints sustain an even greater proportion of the load [10]. Most of the foot injuries reported were presented as blisters and abrasions. The blisters could be due to friction resulting from tight-fitting shoes. Nearly half of the injured participants in 2009 and more than half in 2010 reported heat exhaustion. The lower occurrence of heat exhaustion in the 2009 race may be due to a relatively lower ambient temperature which probably reduced the effects of dehydration and electrolyte imbalance as it had rained heavily the night preceding the event. Running in a marathon has been reported to jeopardize fluid balance and exercise-induced dehydration alters fluid-electrolyte homeostasis, cardiovascular functions and thermal balance [7]. The fact that heat exhaustion was relatively low in 2009 could also have had a bearing on the reduction in the incidence of muscle cramps because of low incidence of dehydration and hyperthermia. Muscle cramps are mostly caused by mineral loss and dehydration as a result of profuse sweating [7].

Cryotherapy was the most utilized single mode of physical treatment. It was used for about one fifth of the injured runners in the two races although most of them had combination treatments which included techniques such as bandaging, cooling, stretching, massage and bandaging among other possible combinations. Previous studies indicate that the application of massage manoeuvres alone after racing events does not appear to alleviate the physiological symptoms of endurance activities such as muscle strength loss, swelling or soreness much faster than the no treatment condition [11,12]. Although this report is not in a position to uphold or dispel this assertion, it is worth noting that for most cases in this study a combination of therapy procedures was utilized thereby improving the chances of alleviating the discomfort felt by the runners.

A major weakness of this report however is that it was not possible to follow up on the participants to establish how many of them developed post-participation health problems such as muscle soreness, swelling and general body aches. It was noted that some injuries and other health problems may not have been reported and this could have resulted in an underestimation of the actual injuries and other health problems. However, all reported cases were recorded and attended to.

## Conclusion

The maiden and the second editions of the Splash 105.5 FM/ICPC Integrity Marathon were successful with respect to care of injured participants. Most injuries were recorded among first-time marathon runners, mostly at the finish line. The part of the body most affected was the thigh and the most reported types of injury and health problem were muscle cramps and heat exhaustion. This report indicates that marathon events can safely take place in a resource-constrained nation and that ample preparation and effective involvement of the physiotherapy team is essential for management of injured runners en route and at the finish line in a marathon. First-time marathon runners should be adequately informed of the predisposition to injury during marathons and adequate body conditioning should be emphasized.

It is recommended that yearly scientific documentation of the injury profile of participants in subsequent marathons be done in order to facilitate adequate planning. Also, a cohort study on the marathoners is recommended to identify injuries and other health problems that may present days immediately after the event and the likely line of management.

## Competing interest

The authors declare that they have no competing interest.

## Authors’ contributions

OOO conceived the idea of the paper and wrote the initial draft of the manuscript. AF revised drafts of the manuscript. Both authors read and approved the final manuscript.

## Pre-publication history

The pre-publication history for this paper can be accessed here:

http://www.biomedcentral.com/2052-1847/5/6/prepub
